# Two-Dimensional Magnetic Field Sensor Based on Silicon Magnetic Sensitive Transistors with Differential Structure

**DOI:** 10.3390/mi8040095

**Published:** 2017-03-23

**Authors:** Xianghong Yang, Xiaofeng Zhao, Yunjia Bai, Meiwei Lv, Dianzhong Wen

**Affiliations:** Key Laboratory of Electronics Engineering, College of Heilongjiang Province, Heilongjiang University, Harbin 150080, China; 2141220@s.hlju.edu.cn (X.Y.); 2161317@s.hlju.edu.cn (Y.B.); 2141200@s.hlju.edu.cn (M.L.); 2141207@s.hlju.edu.cn (D.W.)

**Keywords:** two-dimensional (2D) magnetic field sensor, silicon magnetic sensitive transistor, differential structure, micro-electromechanical systems (MEMS) technology

## Abstract

A two-dimensional (2D) magnetic field sensor consisting of four silicon magnetic sensitive transistors (SMSTs) with similar characteristics is presented in this paper. By use of micro-electromechanical systems (MEMS) and integrated packaging technology, this sensor fabricated by using the silicon wafer with a <100> orientation and high resistivity, was packaged on printed circuit boards (PCBs). In order to detect the magnetic fields in the *x* and *y* axes directions, two of the four SMSTs with opposite magnetic sensitive directions were located along the *x* and −*x* axes directions, symmetrically, and the others were located along the *y* and −*y* axes directions. The experimental results show that when the *V*_CE_ = 10.0 V and *I*_B_ = 6.0 mA, the magnetic sensitivities of the sensor in the *x* and *y* axes directions are 366.0 mV/T and 365.0 mV/T, respectively. It is possible to measure the 2D magnetic field and improve the magnetic sensitivity, significantly.

## 1. Introduction

To the best of our knowledge, with the development of complementary metal-oxide-semiconductor (CMOS) and micro-electromechanical systems (MEMS) technology, magnetic field sensors have been applied in many different fields, such as in the detection of the Earth’s magnetic field and angular position, etc. [1,2,3]. To date, the widely used magnetic sensitivity sensors include Hall sensor, giant magnetoresistance (GMR), magnetic sensitive transistor, and so on [4,5,6]. In 2003, the silicon magnetic sensitive transistor (SMST) with a cubic structure was fabricated by MEMS technology [7,8], achieving a maximum relative magnetic sensitivity of about 22.7%/kG. In 2013, the SMSTs with a differential structure were fabricated by MEMS technology and achieved an absolute magnetic sensitivity of 102.9 mV/kG [9]. In 2015, a two-dimensional (2D) magnetic field sensor based on the operating principle and characteristics of the magnetic sensitive diode (MSD) was proposed [10], exhibiting sensitivities of *S_x_*_B_ = 544 mV/T and *S_y_*_B_ = 498 mV/T in the *x* and *y* directions, respectively. In 2010, in order to improve the efficiency of the magnetic modulating system, a 2D Hall micro-sensor with lower noise and angular positioning function was proposed [11]. In 2013, based on peripheral processing electronics, a new type of 2D differential folded CMOS Hall device with higher accuracy was devised by integrating magneto-transistors in a single chip and connecting a p-substrate to the minimum power supply [12], so as to obviously reduce noise and cross-sensitivity.

In view of the operating principle and characteristics of the SMST, a 2D magnetic field sensor composed of two differential structures with four SMSTs is proposed in this paper. The sensor was studied by using a magnetic field generator system. The detection of two-dimensional magnetic fields is significantly enhanced by using the differential structures and a referable uniform magnetic sensitivity is realized.

## 2. Basic Structure and Operation Principle

### 2.1. Basic Structure

**Figure 1:**
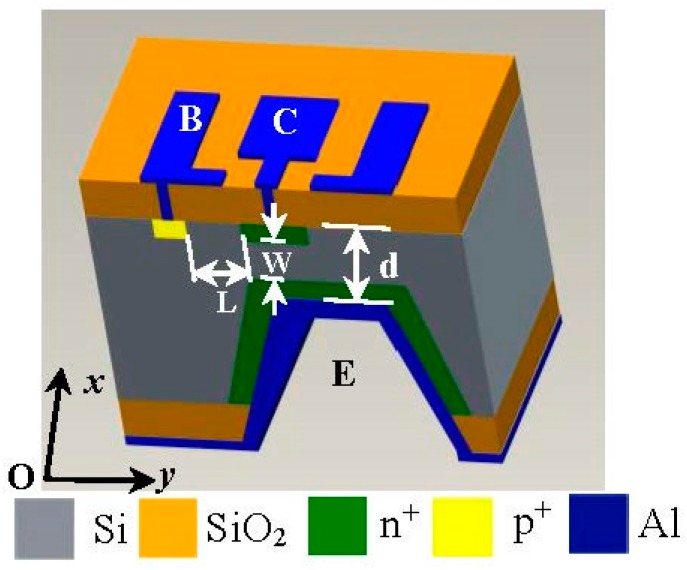
The cubic structure of the silicon magnetic sensitive transistor. E: emitter; B: base; and C: collector.

**Figure 2:**
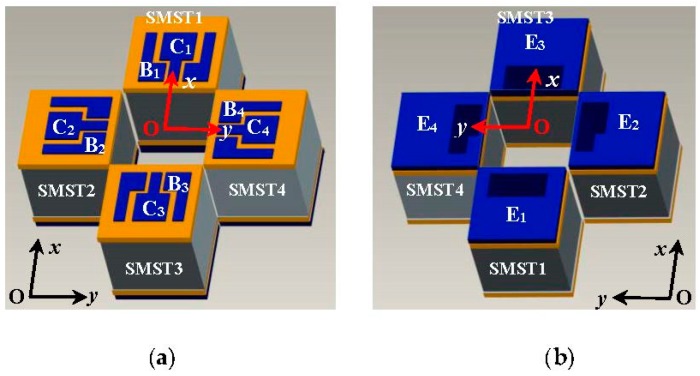
The basic structure of the two-dimensional (2D) magnetic field sensor: (**a**) top view; (**b**) bottom view.

Figure 1 shows the cubic structure of the SMST based on MEMS technology. It has a C-type silicon cup, an emitter (E), a base (B) and a collector (C); the C and B are on top of E. The thickness *d* of the C-type silicon cup diaphragm is 30 µm, and the width *W* and the length *L* of the base region of the SMST are 30 µm and 120 µm, respectively. The cross-section area of the chip is 2000 μm × 2000 μm. As shown in Figure 2, the 2D magnetic field sensor is made up of two differential structures consisting of the four SMSTs with opposite magnetic sensitive directions, where one of the differential structures is constructed by SMST1 and SMST3 located along the *x* and −*x* axes, and another is composed of the SMST2 and SMST4 along the *y* and −*y* axes. The sensor has a common emitter E, four collectors (C_1_, C_2_, C_3_, C_4_), and four bases (B_1_, B_2_, B_3_, B_4_).

### 2.2. Operation Principle

**Figure 3:**
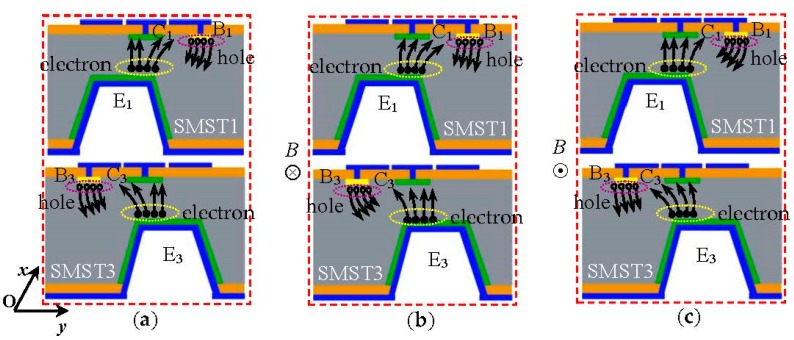
The operation principle of the silicon magnetic sensitive transistor (SMST) differential structure: (**a**) *B* = 0 T; (**b**) *B* > 0 T; (**c**) *B* < 0 T.

**Figure 4:**
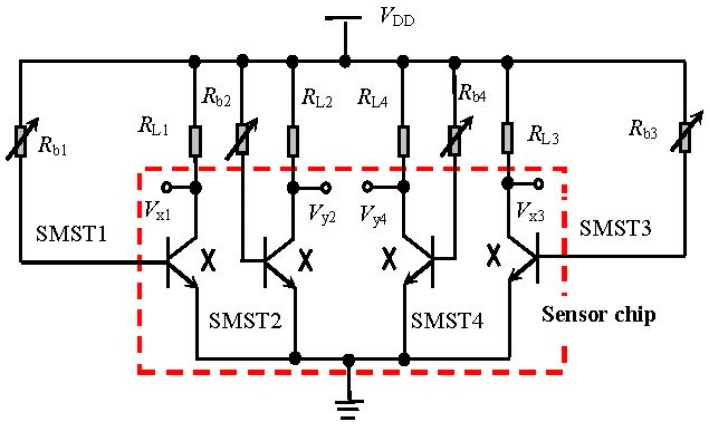
The equivalent circuit of the 2D magnetic field sensor.

Figure 3a–c shows the cross-section views of the SMST1 and SMST3 along opposite magnetic sensitive directions; the operation principle for the 2D magnetic field sensor is discussed on the basis of the carriers deflected by different external magnetic fields. Figure 4 shows the test equivalent circuit of the 2D magnetic field sensor, where *V_x_*_1_, *V_y_*_2_, *V_x_*_3_ and *V_y_*_4_ are the collector voltages for the SMST1, SMST2, SMST3, and SMST4; *R*_L1_, *R*_L2_, *R*_L3_ and *R*_L4_ are the collector load resistances placed between the supply voltage *V*_DD_ and the collectors C_1_, C_2_, C_3_ and C_4_, respectively. *R*_b1_, *R*_b2_, *R*_b3_ and *R*_b4_ are the base resistances placed between the supply voltage *V*_DD_ and the bases B_1_, B_2_, B_3_ and B_4_, respectively. The four integrated packaging SMSTs are given in the dashed square, as shown in Figure 4.

In the ideal case, the two SMSTs along the *x* axis have the same characteristics, in which the deflection of carriers is the same under no external magnetic field, as shown in Figure 3a. The collector output voltage *V_x_* along the *x* axis is:

*V_x_* = *V_x_*_1_ − *V_x_*_3_ = *I*_C03_·*R*_L3_ − *I*_C01_·*R*_L1_ = 0 (1)

where the collector current *I*_C01_ of the SMST1 is equal to the collector current *I*_C03_ of the SMST3.

In contrast, when exerting an external magnetic field *B* parallel to the chip that is along the *x* and −*x* axes respectively, the carriers would be deflected by the Lorentz force as shown in Figure 3b,c. When the magnetic field direction in Figure 3b is defined as positive (*B* > 0 T); the carriers of SMST1 are deflected to a recombination region, as a sequence; *I*_C1_ is decreased and its decrement is Δ*I*_C1_; the collector *I*_C3_ of the SMST3 is increased and its increment is Δ*I*_C3_; the output voltage *V_x_* can be obtained:

*V_x_* = *V_x_*_1_ − *V_x_*_3_ = *I*_C3_·*R*_L3_ − *I*_C1_·*R*_L1_ = Δ*V_x_*_3_ − Δ*V_x_*_1_ (2)

where the values of Δ*V_x_*_1_ and Δ*V_x_*_3_ are the variables of collector output voltages for the SMST1 and SMST3 under the external magnetic field, respectively.

Theoretical analysis dictates that if reversing the external magnetic field as negative (*B* < 0 T), as shown in Figure 3c, *V_x_* can be given:

*V_x_* = *V_x_*_1_ − *V_x_*_3_ = *I*_C3_·*R*_L3_ − *I*_C1_·*R*_L1_ = − (Δ*V_x_*_3_ − Δ*V_x_*_1_) (3)

Moreover, the absolute magnetic sensitivity *S_x_* of the differential structure along the *x* axis can be expressed as:

(4) $$S_{x}=\frac{\left|\Delta V\right|}{B}=\frac{\left|V_{x3}-V_{x1}\right|}{B}=S_{x1}+S_{x3}$$

where *B* is the external magnetic field, *S_x_*_1_ and *S_x_*_3_ are the absolute magnetic sensitivities for the SMST1 and SMST3, respectively. *S_x_* is the sum of the sensitivities of the SMST1 and SMST3, so as to improve the magnetic sensitivity.

The same as above, when there is no external magnetic field along the *y* or −*y* axes, the carriers of the SMST2 and SMST4 are also deflected, and the collector output voltage *V_y_* along the *y* axis is

(5) $$V_{y}=V_{y2}-V_{y4}=I_{C04}\cdot R_{L4}-I_{C02}\cdot R_{L2}=0$$

When an external magnetic field parallel to the chip is applied along the *y* axis direction, the magnetic field is defined as positive, and the carriers of the SMST4 are deflected to the recombination region, therefore *I*_C4_ is decreased and its decrement is Δ*I*_C4_, while the collector *I*_C2_ of the SMST2 is increased and its increment is Δ*I*_C2_, the output voltage *V_y_* can be obtained as:

(6) $$V_{y}=V_{y2}-V_{y4}=I_{C4}\cdot R_{L4}-I_{C2}\cdot R_{L2}=\Delta V_{y4}-\Delta V_{y2}$$

where the values of Δ*V_y_*_2_ and Δ*V_y_*_4_ are variables of the collector output voltages for the SMST2 and SMST4 under the external magnetic field, respectively.

Reversing the external magnetic field as negative, *V_y_* can be given as:

(7) $$V_{y}=V_{y2}-V_{y4}=I_{C4}\cdot R_{L4}-I_{C2}\cdot R_{L2}=-(\Delta V_{y4}-\Delta V_{y2})$$

Moreover, the absolute magnetic sensitivity *S_y_* of the differential structure can be expressed as:

(8) $$S_{y}=\frac{\left|\Delta V\right|}{B}=\frac{\left|V_{y2}-V_{y4}\right|}{B}=S_{y2}+S_{y4}$$

The magnetic sensitivity *S_y_* is also equal to the sum of the sensitivities of the SMST2 and SMST4, therefore the magnetic sensitivity can be improved as well.

## 3. Fabrication Technology

**Figure 5:**
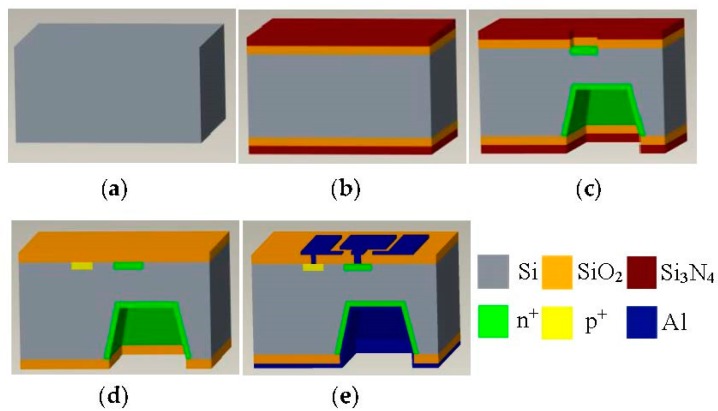
The fabrication technology of the silicon magnetic sensitive transistor. (**a**) Cleaning wafer; (**b**) growing the SiO_2_ layer and depositing the Si_3_N_4_ layer; (**c**) making the collector and the emitter regions by photolithography; (**d**) fabricating the base region by photolithography; (**e**) fabricating the electrodes.

**Figure 6:**
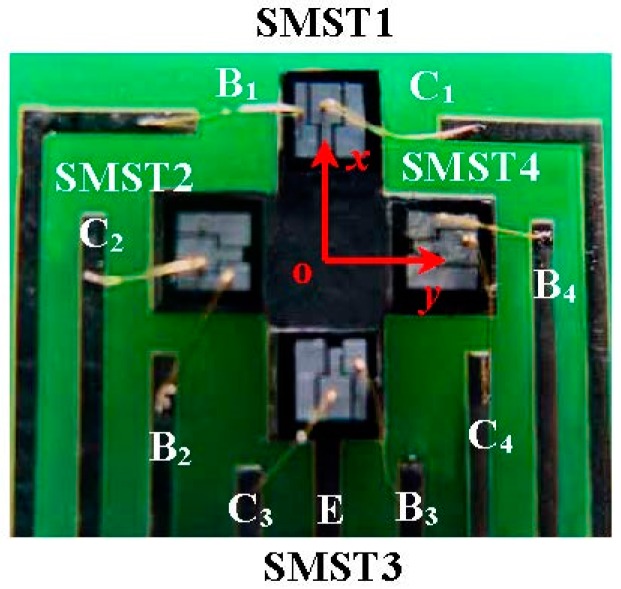
The packaging photograph of the 2D magnetic field sensor.

The main fabrication process of the SMSTs is shown in Figure 5, and the processing steps are as follows: (a) cleaning the p-type silicon wafer with a <100> orientation and high resistivity (ρ = 1000 Ω·cm); (b) growing the SiO_2_ layer (450 nm) by using a thermal oxide method and depositing the Si_3_N_4_ layer (200 nm) by low-pressure chemical vapor deposition (LPCVD) on the double-side of the silicon wafer; (c) etching Si_3_N_4_ and SiO_2_ to form a window of the emitter region by first photolithography, then etching a C-type silicon cup with the silicon diaphragm thickness of 30 μm (emitter regions) by the KOH anisotropic etching technique, fabricating the collector region by second photolithography and forming the collector and the emitter regions by diffusing dense phosphorus; (d) photo-etching the base region and etching the double-sided Si_3_N_4_ layers and the upper-sided SiO_2_ layer after diffusing dense boron, and then depositing the single-side of the SiO_2_ layer; (e) etching contacts of the collector and the base, and depositing an aluminum film on the upper surface by vacuum coating; after that, fabricating the aluminum electrodes of the collector and the base by photography, and then depositing an aluminum film on the bottom to form the aluminum electrode of the emitter and metallizing at 420 °C for a half-hour to form better Ohmic contacts with the base, collector, and emitter. Then, the four chips which have similar characteristics and opposite magnetic sensitive directions are packaged. Figure 6 shows the packaging photograph of the 2D magnetic field sensor based on the SMSTs.

## 4. Results and Discussion

### 4.1. Magnetic Sensitive Characteristics of SMSTs

**Figure 7:**
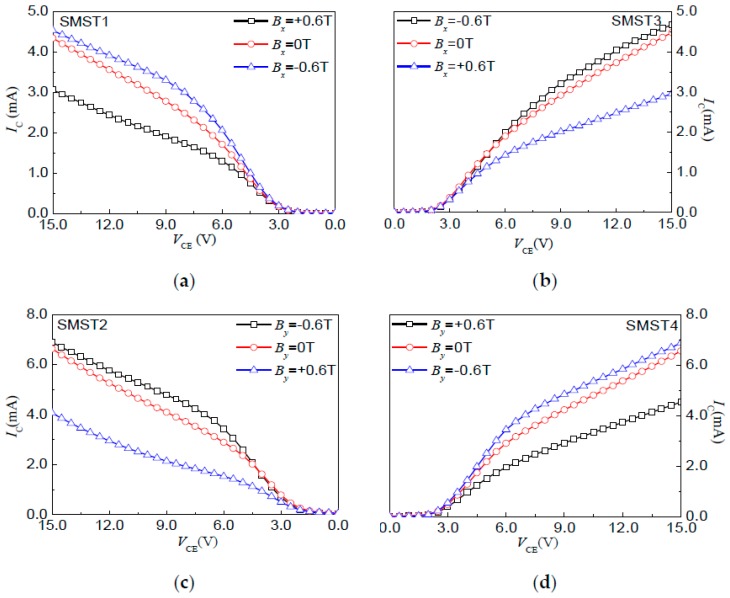
The *I*–*V* characteristic curves of the silicon magnetic sensitive transistor: (**a**) SMST1; (**b**) SMST3; (**c**) SMST2; (**d**) SMST4.

The magnetic sensitive characteristics of the SMSTs were studied by a semiconductor characterization system (4200, KEITHLEY, Cleveland, OH, USA) and a magnetic field generator (CH-100, Cuihaijiacheng Magnetic Technology, Beijing, China) at room temperature. The magnetic sensitive characteristics of the SMST1 and SMST3 were measured under a magnetic field along the *x*-axis direction, and those of the SMST2 and SMST4 were measured under a magnetic field along the *y*-axis direction. When the operating current *I*_B_ = 6.0 mA in a 2D magnetic field range of 0.1 mT–1.2 T, the *I*–*V* characteristic curves of the four SMSTs with a resolution of 1 Gs at *B* = −0.6 T, *B* = 0 T and *B* = 0.6 T are shown in Figure 7, respectively.

**Figure 8:**
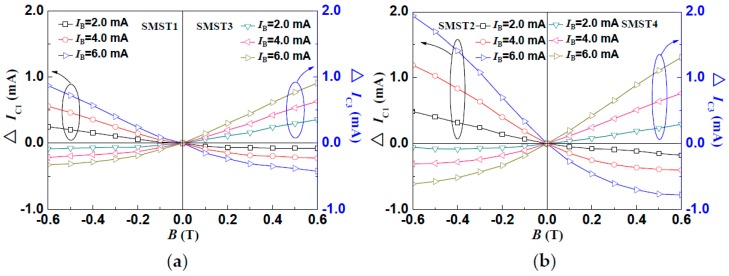
The Δ*I*_C_–*B* characteristics of the silicon magnetic sensitive transistor: (**a**) SMST1 and SMST3; (**b**) SMST2 and SMST4.

The experiment results show that, under a constant external supplied voltage, the measured *I*_C1_ decreases with the increasing magnetic field, and moves towards the positive direction, while *I*_C3_ increases under the same conditions. However, in the subsequent opposite magnetic field direction, the *I*_C1_ increases, and the *I*_C3_ decreases with the increasing magnetic field. Moreover, under the same conditions, the *I*–*V* characteristics of the SMST2 and SMST4 are similar to those of the SMST1 and SMST3, respectively. It indicates that all of the four SMSTs have positive and negative magnetic characteristics. Figure 8a,b shows the relationship curves between the output current (*I*_C1_, *I*_C3_, *I*_C2,_ *I*_C4_) and *B*, where the magnetic field ranges from −0.6 T to 0.6 T with a step of 0.1 T. It shows that the measured *I*_C_ increases not only with a constant operating current but also a constant external magnetic field. When the *V*_CE_ = 10.0 V and *I*_B_ = 6.0 mA, the sensitivities of the SMST1, SMST2, SMST3 and SMST4 are 1.08 mA/T, 1.03 mA/T, 1.89 mA/T and 1.92 mA/T, respectively.

### 4.2. 2D Magnetic Sensitive Characteristics

**Figure 9:**
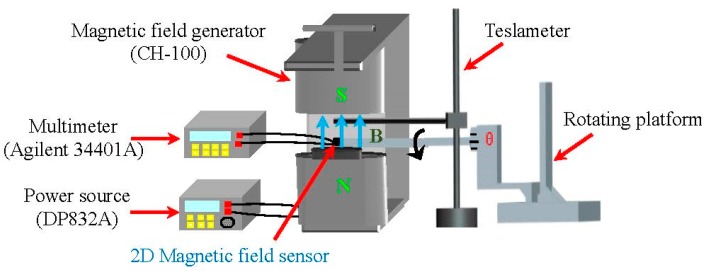
Testing system of the 2D magnetic field sensor.

**Figure 10:**
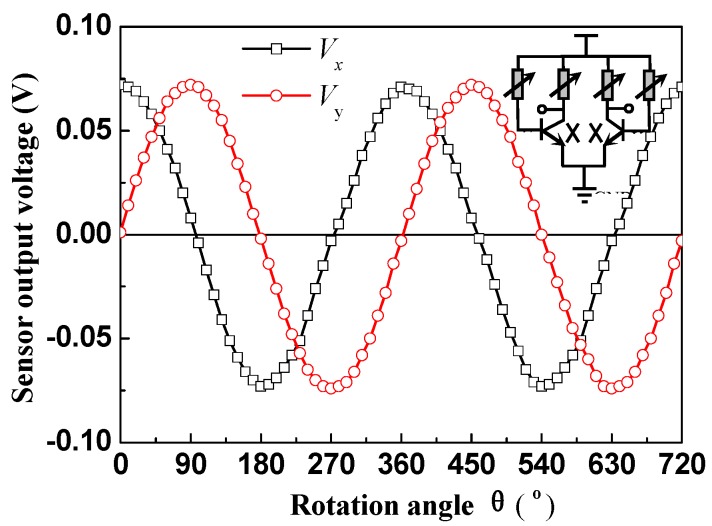
Characteristic curves of the 2D magnetic field sensor at *B* = 0.2 T.

The 2D magnetic sensitive characteristics of the proposed sensor were studied by the rotation platform, magnetic field generator (CH-100), teslameter, multi-meter (Agilent 34401A, Agilent, Santa Clara, CA, USA), and power source (DP832A, RIGOL, Beijng, China), as shown in Figure 9, where the θ is the rotation angle of rotating platform. First, the sensor was fastened on the rotating platform, where the θ is defined as zero when the magnetic induction direction parallel to the sensor surface is along the *x* axis. At room temperature, the relationship of the sensitivities for the 2D magnetic field sensor at about *V_x_*–θ and *V_y_*–θ was studied by every equivalent circuit respectively, as shown in Figure 4. During the test process, to achieve the same sensitivity of the sensors in the two directions, some external resistors were added between the collector and *V*_DD_. When the supply voltage is 10.0 V and the current is 6.0 mA, the characteristic curves of output voltages (*V_x_* and *V_y_*) and θ for the sensor at *B* = 0.2 T are shown in Figure 10.

The results indicate that under a constant external magnetic field *B* and operating voltage, θ = 0°, *V_x_* is nearly the maximum and *V_y_* is very small. With an increasing rotation angle θ, *V_x_* is reduced and *V_y_* is increased, gradually. When θ = 90°, *V_x_* is nearly zero and *V_y_* is close to the maximum. When θ = 180°, *V_x_* is nearly the minimum and *V_y_* is nearly zero. When θ = 270°, *V_x_* is nearly zero and *V_y_* is close to the minimum. When θ = 360°, *V_x_* is nearly the maximum and *V_y_* is close to zero. It shows that the output voltages *V_x_* and *V_y_* of the 2D magnetic field sensor regularly follow as the sinusoidal or cosine functions of the rotation angle θ, therefore the direction and the strength of the two-dimensional magnetic field can be measured by the sensor. When θ is a constant, the absolute values of output voltages (*V_x_* and *V_y_*) increase with the *B*. It indicates that when *V*_DD_ = 10.0 V and *I*_B_ = 6.0 mA, the magnetic sensitivities of the sensor in the *x* and *y* directions are 360.0 mV/T and 365.0 mV/T, respectively. Equation (9) is the average value expression of the output voltage in the whole period.

(9) $${\bar{V}}_{\mathrm{out}}=\frac{1}{n}\sum_{i=1}^{n}V_{\mathrm{out}i}=\frac{1}{n}\sum_{i=1}^{n}\sqrt{{V_{\mathrm{out}xi}}^{2}+\;{V_{\mathrm{out}yi}}^{2}}(i=1,2,3...37)$$

**Figure 11:**
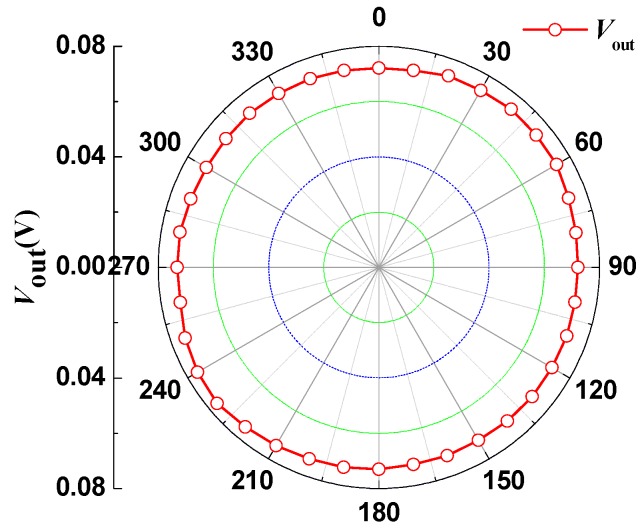
Relationship curves for the output voltage *V*_out_ and θ.

According to Equation (9), under the external magnetic field *B*, the relationship curves of the output voltage *V*_out_ and the rotation angle θ are shown in Figure 11. In the ideal situation, *V*_out_ is a constant and the curve is a circle with a radius of 73 mV. The experiment results show that the output voltage *V*_out_ changes with the rotation angle θ, attributable to the different characteristics of the four SMSTs in the *x* and *y* directions.

In terms of a synthetic analysis of journal articles and experimental results, some designs of 2D magnetic field sensors based on diodes, transistors, and Hall elements are summarized in Table 1. From Table 1, the study of the 2D magnetic field sensor includes Hall components, magnetic diodes and magnetic sensitive transistors, and so on. In this work, a novel 2D magnetic field sensor based on a magnetic sensitive transistor by MEMS technology is designed and fabricated; the magnetic sensitivities in the *x* and *y* directions could approach 366.0 mV/T and 365.0 mV/T, respectively. In particular, a referable uniform sensitivity can be measured along the *x*-axis and *y*-axis. The study on the novel 2D magnetic field sensor helps to provide a new guide for development in the monolithic integrated magnetic field sensor field.

## 5. Conclusions

A new type of 2D magnetic field sensor based on the operating principle and characteristics of the silicon magnetic sensitive transistor (SMST) is proposed in this paper. The sensor was composed of two differential structures with four SMSTs. The experiment results show that when the *V*_CE_ = 10.0 V and *I*_B_ = 6.0 mA, the magnetic sensitivities of the sensor in the *x* and *y* directions reach 366.0 mV/T and 365.0 mV/T, respectively. Based on the differential structure of SMSTs, the 2D magnetic field sensor with a resolution of 1 Gs can measure a 2D magnetic field from 0.1 mT to 1.2 T, not only significantly improving the magnetic sensitivity but also exhibiting a referable uniformity along the *x*-axis and *y*-axis.

## Figures and Tables

**Table 1:** Two-dimensional (2D) magnetic field sensors based on magnetic diodes, transistors and Hall elements. SOS: silicon-on-sapphire; CMOS: complementary metal-oxide-semiconductor; IC: integrated circuit; MEMS: micro-electromechanical systems.

Structure	Technology	Sensitivity	Reference
Magneto-diode	SOS	0.46 μA/mT	[2]
Hall sensor	Simple planar	41 V/AT, 30 V/AT	[5]
Hall sensor	CMOS	19 V/AT, 19 V/AT	[6]
Diode	Bipolar	544 mV/T, 498 mV/T	[10]
Hall device	CMOS	9.564 mV/T	[12]
Transistor a Hall plate	CMOS	30%/T	[13]
Hall element	Bipolar IC	39 V/AT	[14]
Transistor	MEMS	366 mV/T, 365 mV/T	In this work

## References

[1] Almeida M.J., Götze T., Ueberschär O., Matthes P., Müller M., Ecke R., Exner H., Schulz S.E. (2015). Monolithic integration of 2D spin valve magnetic field sensors for angular sensing. Mater. Today Proc..

[2] Roumenin C.S. (1996). 2D magnetodiode sensors based on SOS technology. Sens. Actuators A.

[3] Shapovalov G., Chektybayev B., Sadykov A., Skakov M., Kupishev E. (2016). Experimental measurement of magnetic field null in the vacuum chamber of KTM tokamak based on matrix of 2D Hall sensors. Fusion Eng. Des..

[4] Zhao X.F., Li B.Z., Wen D.Z. (2017). Fabrication technology and characteristics of a magnetic sensitive transistor with nc-Si:H/c-Si heterojunction. Sensors.

[5] Roumenin C.S., Nikolov D., Ivanov A. (2004). A novel parallel-field Hall sensor with low offset and temperature drift 2D integrated magnetometer. Sens. Actuators A.

[6] Lozanova S.A., Roumenin C.S. (2009). A novel magnetometer based on a parallel-field silicon Hall sensor. Procedia Chem..

[7] Zhao X.F., Wen D.Z. (2005). Negative-resistance oscillations characteristics of a new type silicon magnetic sensitive transistor on MEMS. J. Semicond..

[8] Zhao X.F., Wen D.Z. (2006). Fabrication–technology research of new type silicon magnetic-sensitive transistor. Rare Met. Mater. Eng..

[9] Zhao X.F., Wen D.Z., Pan D.Y., Guan H.Y., Lv M.W., Li L. (2013). Differential structure and characteristics of new type silicon magnetic sensitivity transistor. Chin. Phys. Lett..

[10] Zhao X.F., Yang X.H., Yu Y., Wu T., Wen D. (2015). Characteristics of 2D magnetic field sensor based on magnetic sensitivity diodes. Am. Inst. Phys. Adv..

[11] Lozanova S., Roumenin C. (2010). Angular position device with 2D low-noise Hall microsensor. Sens. Actuators A.

[12] Yu C.P., Sung G.M. (2012). Two-dimensional folded CMOS Hall device with interacting lateral magnetotransistor and magnetoresistor. Sens. Actuators A.

[13] Janković N., Pantić D., Batcup S., Igic P. (2012). A lateral double-diffused magnetic sensitive metal-oxide-semiconductor field-effect transistor with integrated n-type Hall plate. Appl. Phys. Lett..

[14] Lozanova S.V., Noykov S.A., Ivanov A.J., Roumenin C.S. (2015). 2D in-plane Hall sensing based on a new microdevice couple concept. Procedia Eng..

